# Is there a cloud in the silver lining for imatinib?

**DOI:** 10.1038/sj.bjc.6600828

**Published:** 2003-04-01

**Authors:** S C Paterson, K D Smith, T L Holyoake, H G Jørgensen

**Affiliations:** 1 Department of Bioscience, Royal College, 204 George Street, University of Strathclyde, Glasgow G1 1XW, UK; 2 Haemato-Oncology Section, Division of Cancer Sciences and Molecular Pathology, University of Glasgow, Level 3 Queen Elizabeth Building, Royal Infirmary, 10 Alexandra Parade, Glasgow G31 2ER, UK

**Keywords:** CML, imatinib, drug resistance

## Abstract

Imatinib mesylate (Gleevec® or Glivec®), a small molecule tyrosine kinase inhibitor for the treatment of chronic myeloid leukaemia, has been said to herald the dawn of a new era of rationally designed, molecularly targeted oncotherapy. Lurking on the same new horizon, however, is the age-old spectre of drug resistance. This review sets the intoxicating clinical perspective against the more sobering laboratory evidence of such divergent mechanisms of imatinib resistance as gene amplification and stem cell quiescence. Polychemotherapy has already been considered to combat resistance, but a more innovative, as yet unformulated, approach may be advocated.

Chronic myeloid leukaemia (CML), a myeloproliferative disorder of haemopoietic stem cell origin, is characterised by the t(9;22) reciprocal translocation giving rise to a shortened chromosome 22, the so-called Philadelphia (Ph) chromosome. The resultant novel fusion oncoprotein, BCR-ABL, has constitutive tyrosine kinase activity and is considered causative in the disease. BCR-ABL tyrosine kinase impinges on a number of downstream signalling pathways resulting in the disruption of normal control of cellular events such as proliferation, adhesion to bone marrow stroma and apoptosis (reviewed in Sawyers, 1999). As BCR-ABL protein expression is disease specific, and is present in 95% of CML cases, it was a logical target for rationally designed therapy. Indeed, signal transduction inhibitor 571 (STI571) emerged as a fastidious BCR-ABL antagonist (Druker *et al*, 1996) inducing remarkable haematological and cytogenetic remissions in interferon-intolerant, -refractory or -resistant CML patients in the stable chronic phase of the disease (Druker *et al*, 2001). STI571 is now marketed as Glivec® (Gleevec® in USA); however, the current focus of many laboratories is to elucidate mechanisms of resistance which may emerge to the drug. In modelling such events, the hope would be that clinicians could deliver a pre-emptive strike against what is proving to be a tenacious and fascinating adversary in BCR-ABL.

## DISEASE CHARACTERISTICS AND TREATMENT

CML is a triphasic disease, beginning with a relatively innocuous chronic phase (CP), in which 50% of patients may be asymptomatic, which lasts on average 4–5 years, progressing into accelerated phase (AP, 6–18 months duration) and terminating in fatal blast crisis (BC, 6 months) (Sawyers, 1999). As CML progresses to BC, the malignant clone acquires additional genetic mutations, including trisomy 8, isochromosome i (17q), trisomy 19 and double Ph, in the majority of patients and so the therapeutic window, in terms of curative intent, is present only in the early course of the disease. Moreover, mutations, deletions and rearrangements of tumour-suppressor genes often arise.

In the 1950s, radiotherapy, splenectomy or chemotherapy were the mainstay of treatment for CML. However, neither busulfan nor hydroxyurea, antimetabolites still in use today, are more than palliative as they fail to induce cytogenetic remission (CyR). Interferon alpha (IFN-*α*), however, can induce a major CyR (⩽35% Ph^+^ metaphases) in up to 38% of patients, although its use is associated with severe side effects, with the majority of patients requiring dose reduction, or, indeed, cessation. IFN-*α* may be coadministered with low-dose cytarabine, with better response rates and survival being observed (Guilhot *et al*, 1997); however, the combination tends to cause greater gastrointestinal tract and mucosal toxicity. Another drug used in combination with IFN-*α* is homoharringtonine (O'Brien *et al*, 2002), which is a plant alkaloid with comparable activity. Recently, a pegylated form of IFN has entered clinical trials (Talpaz *et al*, 2001). Pegylated IFN-*α* has longer residence time in the body, hence a weekly, rather than daily, subcutaneous dosing regimen. Of 27 CML patients intolerant or resistant to IFN-*α* entered into a Phase I trial, pegylated IFN-*α* induced a complete CyR in two of 19 patients with active disease. Of the remaining eight patients already in complete haematological remission (CHR), four achieved a complete, and three a partial, CyR (Talpaz *et al*, 2001).

To date, only allogeneic bone marrow (allo-BMT) or peripheral blood stem cell (PBSC) transplants offered any hope of a cure for CML. Transplants cannot be offered to all patients, however, owing to the lack of donor availability. Further, the age-related increased risk of undergoing a transplant, along with greater likelihood of concomitant disease such as diabetes or heart disease, mean that in general this procedure is not performed on patients over the age of 55 years. Thus, as p210^BCR-ABL^ is present in 95% of CML patients, is thought causative in the disease, and is constitutively active, a drug specifically designed to target its tyrosine kinase activity would offer the best hope for a nontransplant cure.

## IMATINIB MESYLATE

**Figure 1:**
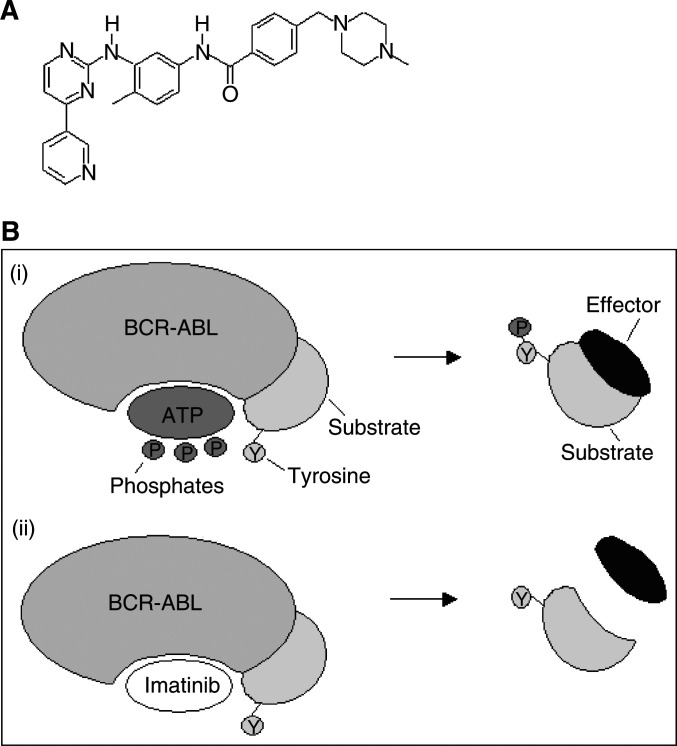
(**A**) Molecular structure of imatinib. (**B**) Mode of action of imatinib. (i) ATP binds to BCR/ABL and phosphorylates a tyrosine (Y) residue of the substrate. The substrate can then bind to an effector molecule triggering the cellular response. (ii) Imatinib binds to BCR/ABL blocking the binding of ATP, thus the substrate tyrosine molecule is not phosphorylated and cannot in turn bind and activate the effector molecule.

One such molecularly targeted small molecule inhibitor is imatinib mesylate (formerly STI571) a 2-phenylamino pyrimidine compound (Figure 1A

). The BCR-ABL tyrosine kinase requires phosphate from ATP to phosphorylate its substrate molecules. Imatinib occupies the ATP-binding site on BCR-ABL, thus preventing substrate phosphorylation (Figure 1B). Imatinib is not specific to BCR-ABL as it also interacts with the cell surface receptor c-KIT in addition to regulating the kinase activity of the platelet-derived growth factor receptor (PDGFR) (Druker *et al*, 1996). Indeed, imatinib has been found to inhibit phosphorylation of c-KIT implicated in gastrointestinal stromal tumours and now provides a new therapeutic option for this most common of all gastrointestinal tract mesenchymal tumours (Demetri *et al*, 2002).

Imatinib has a low toxicity profile compared to many other cytotoxic agents, indeed a maximum tolerated dose was not reached in Phase I/II trials in CML (Druker *et al*, 2001). The most common side effects were low-grade nausea, vomiting and other gastrointestinal side effects including diarrhoea, reflux and taste disturbance, as well as oedema (in particular periorbital), and skin rashes. In a small proportion of patients, these toxicities significantly affected their quality of life. For those patients in BC taking imatinib at the higher dose of 800–1000 mg day^−1^, more toxic side effects (Grade 3–4 thrombocytopenia and neutropenia) were evident. It should be remembered that pancytopenia is more commonly seen in AP/BC CML reflecting the suppression of normal haemopoiesis at this stage of the disease. Platelet and WBC counts may take time to normalise on imatinib; however, if counts do not recover adequately, these effects can be minimised by imatinib dose reduction or temporary cessation of treatment.

Numerous clinical trials have been conducted using imatinib as a treatment for CML (summarised in Table 1

**Table 1:** Comparison of responses to imatinib therapy in CML

**Study**	**Disease phase**	**No. of patients**	**Imatinib (daily dose)**	**Complete HR (%)**	a**MCyR (%)**	**Complete CyR (%)**
bIRIS (imatinib) Guilhot *et al* (2002)^d^	cCP-Dx	553	400 mg	94	83	68
*IRIS (IFN-α)*	*CP-Dx*	*553*	*5 MIU m*^*−2*^ *IFN + 20 mg m*^*−2*^ *cytarabine*	*55*	*20*	7
Druker *et al* (2001)	CP	54	>300 mg	98	54	13
Kantarjian *et al* (2002c)	Late CP	454	400 mg	95	76	41
Kantarjian *et al* (2002b)	AP	200	400–600 mg	80	45	24
Talpaz *et al* (2002)	AP	181	400–600 mg	53	48	17
Kantarjian *et al* (2002a)	BC	75	300–1000 mg	21	11	6
Sawyers *et al* (2002)	BC	229	400–600 mg	15	31	7

a MCyR=major cytogenetic response defined as complete (0% Ph^+^) or partial (1–35% Ph^+^).

b IRIS=International Randomized IFN vs. STI571 study.

c CP-Dx=newly diagnosed chronic phase.

d And personal communication from Dr Stephen O'Brien, Royal Victoria Infirmary, Newcastle.

, Druker *et al*, 2001; Guilhot *et al*, 2002; Kantarjian *et al*, 2002a,2002b,2002c; Sawyers *et al*, 2002; Talpaz *et al*, 2002). The published results of these trials have been very encouraging, and would argue for the need for commencement of imatinib therapy following diagnosis to elicit durable responses. Loss of imatinib-induced remissions is presumably because of emerging drug resistance. BCR-ABL reactivation can arise through gene amplification, resulting in higher levels of BCR-ABL protein (Le Coutre *et al*, 2000; Mahon *et al*, 2000; Weisberg and Griffin, 2000; Gorre *et al*, 2001). Alternatively, a gene mutation in the ATP-binding pocket on BCR-ABL, which is the site of action of imatinib, results in the inability of imatinib to competitively bind out ATP (Gorre *et al*, 2001), although other mechanisms of resistance may operate.

## MECHANISMS OF RESISTANCE TO IMATINIB IN Ph^+^ CELLS

### Gene amplification

It has been reported that in a proportion of CML BC-derived cell lines, grown *in vitro* in the continuous presence of imatinib, *BCR-ABL* gene amplification is observed (Le Coutre *et al*, 2000; Mahon *et al*, 2000; Weisberg and Griffin, 2000). Overexpression of BCR-ABL protein has been identified in *BCR-ABL* transfected murine Ba/F3 cells as well as in human LAMA84 and AR230 CML cell lines (Mahon *et al*, 2000). The exact mechanism controlling this enhanced *BCR-ABL* gene transcription and translation remains uncertain, although it is not at the level of the *BCR* gene promoter. If this were the case, the native *BCR* gene on the unaffected chromosome 22 would also be expressed at increased levels in resistant cells. In fact, *BCR* expression was much reduced in the LAMA84-resistant cells as well as in the AR230-resistant cells (Mahon *et al*, 2000). Since BCR protein has been proposed as a negative regulator of BCR-ABL (Wu *et al*, 1999), low BCR levels would compound the insensitivity to imatinib. Le Coutre *et al* (2000) demonstrated *BCR-ABL* mRNA transcription to be 4.6 times higher in their LAMA84-resistant cell line than in the parental imatinib-sensitive cell line. Further, using FISH, the LAMA84R cells were found to harbour ⩾15 *BCR-ABL* gene copies per cell. This study provides further compelling evidence implicating gene amplification in resistance to imatinib.

To determine whether the gene amplification phenomenon occurred in patients, clinical samples from CML patients who relapsed after initially responding to imatinib, were analysed (Gorre *et al*, 2001). In three of 11 samples, multiple copies of the *BCR-ABL* gene were identified in interphase nuclei by FISH (two of three were myeloid BC, the other lymphoid BC). Rather than an overexpressed subclone attaining a relative growth advantage, it has been hypothesised that imatinib itself may have initiated the *BCR-ABL* gene amplification. For example, in one of these three patients who received alternative treatment for relapsed leukaemia, it was no longer possible, 4 weeks after cessation of imatinib therapy, to detect *BCR-ABL* gene amplification. While these are interesting observations, the number of patients in this cohort was small, hence the findings may not be truly representative of all CML BC patients, nor of CML which progresses from CP. Interestingly, Tipping *et al* could restore sensitivity of a LAMA84-resistant cell line to imatinib after 2 months withdrawal from continuous exposure to the drug. It was surmised that a subgroup of resistant patients may benefit from interruption, followed by resumption at a later date, of therapy (Tipping *et al*, 2001).

### Mutations in ATP-binding pocket

Another mechanism underlying imatinib resistance is point mutations in the *BCR-ABL* gene altering the conformation of the ATP-binding pocket such that imatinib no longer has affinity. Analysis of the 579-base pair region, which gives rise to the ATP-binding pocket and the activation loop of the kinase domain on BCR-ABL, revealed in six of nine patients a C→T change at nucleotide 944 (Gorre *et al*, 2001). The latter transition corresponds to an isoleucine substitution for threonine at position 315 (T315I) in the ATP-binding pocket, which does not inhibit ATP binding. Such a change, however, would inhibit drug binding owing to steric hindrance and loss of critical hydrogen bond formation. Von Bubnoff *et al* (2002) have analysed a group of eight patients with Ph^+^ leukaemias, of which two were CML in BC or AP/BC. This group also detected the T315I in one patient, as well as reporting four novel point mutations all located within the drug-binding pocket on BCR-ABL. Branford *et al* (2002) found that nine of 12 CML patients resistant to imatinib had a mutation within the ATP-binding region of BCR-ABL. A range of mutations was observed, including T315I. Roumiantsev *et al* (2002) found that the Y253F mutation in the Abl kinase domain conferred intermediate resistance to imatinib, both *in vitro* and *in vivo*, relative to the T315I mutation.

However, the clinical significance of the T315I mutation to imatinib resistance was questioned by the studies of Barthe *et al* (2001) and Hochhaus *et al* (2001). The former group found no C→T transition, and only one G→A mutation in their analysis of 12 patients, while the latter study identified only one A→T mutation in a cohort of 32 patients. Mahon *et al* (2000) studied the Ph^+^ imatinib resistant-cell lines K562-R and AR230-R, but found no mutations in the ATP binding pocket. Similarly, Le Coutre *et al* (2000) identified no mutations in their LAMA84R cell line. It should be borne in mind, however, the likely range of mutations that will be observed in populations of different genetic background (Barthe *et al*, 2001).

While there is now some consensus regarding the particular mutations detected, the reported incidence of each mutation in patients is variable. The most recent data are more relevant to the clinic since the cells used in these analyses were procured from patients, compared to previous studies employing cell lines (Le Coutre *et al*, 2000; Mahon *et al*, 2000).

### Alpha-1-acid glycoprotein (AGP)

Alpha-1-acid glycoprotein (AGP), an extensively glycosylated plasma protein and known binder of drugs (Fournier *et al*, 2000), changes both quantitatively and qualitatively (in terms of glycosylation) in response to inflammation and disease, such as cancer. In a recent study by Jørgensen *et al* (2002b) investigating a possible link between decreased imatinib efficacy and AGP-drug binding, AGP levels were found to be significantly raised in CML patients at all stages of disease compared to normal controls. Normal AGP, even at up to seven times the normal population average concentration of 0.77 mg ml^−1^ (Blain *et al*, 1985), had no influence on the effect of imatinib on the Ph^+^ K562 BC cell line. AGP isolated from individual CML patients did not confer any loss of function of imatinib in the assay. Further, in a direct binding assay whereby any AGP-imatinib interaction is detected as fluorescence quenching, imatinib did not bind normal AGP at clinically relevant concentrations of the drug (5 *μ*M) (Jørgensen *et al*, 2002c). Gorre *et al* (2001) clearly demonstrated cell intrinsic mechanisms of resistance to Glivec® whereby cells taken from relapsing patients showed reduced sensitivity to the drug with evidence of acquired molecular changes such as gene mutation or amplification.

However, Gambacorti-Passerini *et al*, 2000 (Le Coutre *et al*, 2002) argue for a role for AGP-imatinib binding as a mechanism of drug resistance. To overcome their observed binding of normal AGP (which by definition will have a different glycosylation pattern from CML-derived AGP and be of limited clinical relevance) to imatinib, it was suggested using clindamycin or erythromycin concomitantly, since the antibiotics purportedly compete with imatinib for AGP-binding sites. Larghero *et al* (2001) found whole sera from BC CML patients, comprising AGP up to 3 mg ml^−1^ and numerous other drug-binding candidates, to prevent K562 cell death *in vitro* when incubated with up to 10 *μ*M imatinib. Reportedly, erythromycin addition reversed the effect of AGP. The fact that all the patients in BC failed to respond to imatinib was attributed to the high levels of AGP, while those in the early phases of the disease with lower AGP levels responded to the drug. It was noted in a cohort of 39 patients (Le Coutre *et al*, 2002) that AGP levels were related to rate of response to imatinib; however, the effectiveness of the drug was unaltered by AGP concentration. None of the studies supporting AGP-imatinib binding convincingly distinguish ‘cause and effect’. It has not been proven definitively that an elevated AGP plasma concentration is the direct cause of imatinib resistance as opposed to simply being a surrogate marker for disease progression.

### Quiescent stem cells

To further add to the mechanisms of resistance to imatinib, results from a study by Graham *et al* (2002) stress the importance of quiescent Ph^+^ stem cells. These cells, which can be isolated from all CML patients in CP, are phenotypically primitive expressing the stem cell marker, CD34, and are part of the leukaemic clone being *BCR-ABL*^*+*^ by RT-PCR and Ph^+^ by FISH. While the cells are out of cycle, they have been found to be resistant to the anti-leukaemic effects of imatinib. This would be problematic in patients in whom a molecular remission has been induced by imatinib, which is then discontinued. If quiescent Ph^+^ stem cells persist, which retain the potential to spontaneously enter a proliferating state, the leukaemic clone would then be re-established and CML would recur. In this scenario, continued treatment, even in the minimal residual disease scenario, would be required to target newly emerging leukaemic cells. From a pharmacoeconomic point of view, however, it would be preferable to completely eradicate the Ph^+^ clone enabling discontinuation of therapy. Furthermore, a maintenance strategy runs the risk of forcing the emergence of truly resistant clones, that is, resistant through additional mechanisms other than by virtue of their quiescence.

### Multidrug resistance protein

Resistance of certain cancer cells to drugs such as anthracycline or vinca alkaloids can be caused by an increase in the expression of the multidrug resistance-1 (*MDR-1*) gene (Brinkmann *et al*, 2001). This gene encodes P-glycoprotein (Pgp), a member of the ATP-binding cassette (ABC) protein family, which function to remove toxic molecules from cells. The imatinib-resistant leukaemic cell line LAMA84R was observed to express Pgp at higher levels than the sensitive cell line (Mahon *et al*, 2000). Furthermore, cells taken from patients in BC were found to express Pgp only when the patients had relapsed, and not when they were responding to therapy (Kuwazuru *et al*, 1990). Recent results have indicated that there is a correlation between *MDR-1* gene expression and relapse of patients taking imatinib (Deininger *et al*, 2001). Higher levels of *MDR-1* are observed at the time of relapse compared to levels pre-therapy. While the leukaemic cells may themselves have increased numbers of such xenobiotic transporters, Pgp is also located on the epithelial cells of the gastrointestinal tract. This may decrease upper gastrointestinal absorption of orally administered drugs, such as imatinib, resulting in yet another potential method of resistance. Furthermore, single nucleotide polymorphisms in the *MDR-1* gene may result in altered affinity for cytotoxic drugs, possibly explaining imatinib insensitivity in some patients (Brinkmann *et al*, 2001).

## CONCLUSIONS AND FUTURE PERSPECTIVES

In summary, it is becoming apparent that a variety of mechanisms may result in resistance to imatinib, and indeed some patients may exhibit more than one mode of resistance. In order to combat this, combinatorial therapies against proliferating Ph^+^ cells, and attempts to eradicate quiescent leukaemic stem cells (Jørgensen *et al*, 2002a), will be of great importance in treatment of CML. Combinations of imatinib with IFN-*α* or vincristine (Kano *et al*, 2001); daunorubicin or cytarabine arabinoside (Thiesing *et al*, 2000); or more novel compounds such as the Janus kinase 2 (JAK2) inhibitor AG490 (Sun *et al*, 2001); or farnesyl transferase inhibitors (FTI) (Hoover *et al*, 2002) including SCH66336, may synergistically enhance the effect of imatinib alone. It has yet to be shown if improved survival rates may be achieved by prescribing imatinib along with additional agents in relapsing CML patients. However, pre-emptive combinatorial therapies may prove to avert, or at least delay resistance to imatinib therapy. In addition to selecting a synergistic partner for imatinib, dose scheduling must also be considered as it has also been reported that imatinib sensitivity may be restored in certain drug-resistant CML cells (Tipping *et al*, 2001). It appears to be beneficial to stop treatment with imatinib temporarily and resume at a later date, at which point the CML cells appear sensitive to the drug once again. This phenomenon may be explained by the lack of selection pressure for BCR-ABL imposed on CML cells by imatinib.

Clearly, there are still advances to be made towards the nontransplant cure of CML, but given the unprecedented rapid regulatory approval given to imatinib by the Food and Drug Administration (FDA), further developments of safe and effective, rationally designed drugs should be available to patients quicker than ever before.
